# Prevalence and epidemiology of presumptive cerebral microbleeds in a population of 747 dogs undergoing brain MRI: a retrospective study

**DOI:** 10.1371/journal.pone.0332658

**Published:** 2025-10-10

**Authors:** Cássia Maria Molinaro Coelho, Gabriela Wacheleski Brock, Roberta Amaral Ferraz Barros, Alex Gradowski Adeodato, Mauro Caldas Martins, Tais Ferreira Guimarães, Gabriela Carvalhaes Potgieter, Lucas Rego Ramos, Marcos Fabio DosSantos

**Affiliations:** 1 Postgraduate Program in Veterinary Medicine, Federal Rural University of Rio de Janeiro (UFRRJ), Brazil; 2 Department of Veterinary Medicine and Surgery, Veterinary Institute, Federal Rural University of Rio de Janeiro (UFRRJ), Brazil; 3 Veterinary Clinic CRV Imagem, Rio de Janeiro, Brazil; 4 Laboratory of Cell Morphogenesis, Biomedical Sciences Institute, Federal University of Rio de Janeiro (UFRJ), Brazil; Medical Center - University of Freiburg, GERMANY

## Abstract

Current advances in veterinary medicine have led to a significant increase in the longevity of dogs, and age-related brain changes have become more recognized, such as cerebral microbleeds (CMBs). However, few studies have described their occurrence and epidemiology in dogs. This retrospective study describes the signalment and MRI findings in dogs with presumptive CMBs (pCMBs) and their association with concurrent age-related changes. A database of exams obtained from a veterinary MRI diagnostic center was reviewed. Signalment information such as breed, age, sex, and concurrent MRI findings were evaluated and compared between groups. A total of 747 dogs that underwent 1.5T brain MRI with standard sequences (T2, FLAIR, T1, and T2*) were included. A total of 142 dogs (19%) met the inclusion criteria for pCMBs. The prevalence of pCMBs increased with age, especially in those older than ten years. Small breed dogs were significantly more affected than large-breed dogs. Female dogs showed higher prevalence than males, likely related to longer lifespan. Most dogs had multiple pCMBs (62%), mainly with a lobar distribution (57.7%). Brain atrophy was significantly identified concurrently with pCMBs in 61.3% of the dogs. The results of the current study support that pCMBs constitute a common age-related MRI finding in small-breed dogs and females, frequently associated with brain atrophy.

## Introduction

Current advances in veterinary medicine, along with the perception of domestic animals as family members, have led to a significant increase in the lifespan of dogs. As longevity increases, age-related brain changes become more widely recognized. However, as in humans, there is considerable overlap between normal aging and neuropathological changes, and studies have been developed to better understand these differences. Typical Magnetic resonance imaging (MRI) findings in humans and dogs are brain atrophy, cerebral microbleeds (CMBs), as well as white matter lesions [1–4].

In veterinary medicine, several studies have investigated age-related brain changes in dogs. Hasegawa et al. [1] first described brain atrophy measurements in dogs with cognitive dysfunction, while Kimotsuki et al. [2] documented age-related morphological changes in beagle dogs. More recently, studies have focused on specific lesions such as white matter hyperintensities [4] and cerebral microbleeds [3,5]. However, comprehensive epidemiological studies of presumptive cerebral microbleeds in large canine populations remain limited.

CMBs are small areas of round/oval-shaped intraparenchymal signal voids, best distinguished on T2*-weighted gradient-recalled echo (GRE) or susceptibility-weighted imaging (SWI) [6]. Such lesions correspond to leakage of blood products into perivascular tissues as demonstrated in histopathological exams [7,8].

In humans, CMBs are commonly found in healthy elderly populations, with an incidence of 15–21%, and both the prevalence and the number of lesions increase with age [9,10]. Several studies have demonstrated that CMBs may not be a normal consequence of aging and have been associated with various diseases. They are considered risk factors for intracranial hemorrhage and ischemic stroke [11–15]. CMBs are mainly related to hypertensive arteriopathy (HA) and cerebral amyloid angiopathy (CAA). Their distribution within the brain differs, with strictly lobar and deep CMBs mostly consistent with CAA and HA, respectively [16,17].

Despite being widely recognized in humans, few studies have described the occurrence and epidemiology of presumptive pCMBs in dogs [3,5,18]. The most comprehensive study to date by Kerwin et al. [3] associated the presence of these lesions with a shorter life expectancy. However, the major causes and clinical relevance of pCMBs still need to be explored in depth which led the authors to conduce this study to contribute to the scientific literature related to this topic.

This study aims to describe the MRI findings, number, and distribution of pCMBs in a large population of dogs (747) undergoing brain MRI, as well as their associated epidemiological factors, including breed-specific prevalences and sex-related differences. Additionally, it seeks to correlate the prevalence of pCMBs with other intracranial abnormalities and age-related change. We also aim to compare our findings with the existing veterinary literature, particularly the study by Kerwin et al. [3], to better understand geographic and population differences in pCMB prevalence.

## Materials and methods

This is a retrospective study. The MRI database from a Veterinary Diagnostic Center (CRV Imagem, Rio de Janeiro, Brazil) was reviewed between January 2019 and September 2020. All dogs that underwent brain MRI (1.5-Tesla unit, Siemens Magnetom Essenza, Germany) were included in the study and had been scanned following the standard sequence, as follows: T2-weighted (T2W) spin echo transverse, sagittal, and dorsal plane images, T2*-weighted (T2W) gradient-recalled echo (GRE) transverse images, T2-weighted fluid-attenuated inversion recovery (FLAIR) transverse images, and T1-weighted (pre- and post-contrast) transverse images (all sequences and settings are summarized in Appendix 1). Exclusion criteria included the absence of a T2W GRE sequence, incomplete medical records, and follow-ups of previous MRI exams (e.g., only the initial MRI study was included if a dog had more than one MRI study). Dogs with evidence of pCMBs were included in the pCMB group; the remaining dogs within the whole sample population were used as control cases.

Signalment information was retrieved from the electronic medical database (Mediclinic RIS system) for all dogs, including breed, age, and sex. All breed dogs except mixed breed were classified as small breed (<15 kg), medium breed (15–30 kg), or large breed (>30 kg), depending on the standard weight according to kennel club guidelines (American Kennel Club). Mixed breeds were grouped as one category and were not subdivided by size. Cases were also categorized according to their age: young (<2 years), adult (2–9 years), and elderly (>9 years).

The criteria for pCMBs identification were used based on previous publications [5,6] and included: presence of small round/ovoid signal void with blooming on T2W gradient echo images; absence of T1 and T2 hyperintensity of these lesions; surrounded by brain parenchyma; maximum diameter <5.7mm on T2W gradient echo; no history of trauma. It is important to note that, since the diagnoses could not be confirmed by histopathological exams, these can be considered “MRI-based presumptive diagnoses (as previously defined),” as detailed in the limitations section.

In dogs with MRI features of pCMBs, each lesion identified was quantified (n = 1, between 2–5, n= > 5) based on transverse T2*W GRE sequence and recorded according to their anatomic location, which included: cerebral cortex, basal nuclei, and surrounding white matter, thalamus, brainstem (midbrain, pons and medulla oblongata) and cerebellum. Lesions were also classified based on their distribution into “lobar” (pCMBs restricted to gray matter and subcortical white matter of the cerebral cortex or cerebellum) and “deep” (lesions in the basal nuclei and surrounding white matter, thalamus, and brainstem). Lesions distributed in both lobar and deep brain regions were considered “mixed location”. All MR images were reviewed by two radiologists (M.C.M. [Board certified by the Brazilian Association of Veterinary Radiologists (ABRV) since 2019] and T.F.G. −13 years of experience).

Other MRI findings were recorded for all dogs. Two types of evaluations were performed: (1) concurrent intracranial abnormalities identified based on the original MRI diagnosis reports, and (2) specific re-evaluation of brain atrophy features performed for this study. Concurrent intracranial abnormalities were retrieved from the original radiology reports and classified based on established diagnostic criteria. In addition, the diagnostic criteria of each disease were established according to the typical imaging features described in literature [19], along with signalment characteristics such as age, breed, history, clinical signs, and evolution. Again, it is important to emphasize that considering the impossibility of corroborating the diagnoses through histopathological evaluation, these can be considered “MRI-based presumptive diagnoses (as previously defined),” as noted in the study limitations. They were classified as brain atrophy, cerebrovascular disease (ischemic or hemorrhagic), meningoencephalitis, intracranial neoplasia (intra- or extra-axial), other abnormalities, or normal MRI.

Brain atrophy was specifically re-evaluated for this study based on characteristic MRI features reported in previous studies [1,2,20], which include enlargement of the ventricular system, widening of the cerebral sulci, and smaller interthalamic adhesion. Interthalamic adhesion height was measured at its maximum vertical thickness on transverse T2-weighted images as previously defined [1]. The predictive characteristics of brain atrophy, including enlargement of the ventricular system, widening of the cerebral sulci and size of the interthalamic adhesion were rigorously examined in each MRI exam and described as findings either associated with or independent of other conditions (e.g.,: cerebrovascular disease, intracranial neoplasia, inflammatory conditions, and/or CMB).

A separate analysis of age-related MRI changes was performed specifically in elderly dogs (≧9 years) from both groups (pCMB and control). This represents a secondary analysis focused solely on the elderly population. For this specific age-related analysis, elderly dogs (≧9 years) were evaluated separately from the main study population. The presence of brain atrophy (with the interthalamic adhesion height measurement) as well as white matter lesions (identified as bilateral symmetrical, periventricular white matter T2 and FLAIR hyperintensities), suggestive of leukoaraiosis [4] were recorded.

Statistical analyses were performed using commercial software packages (e.g., SigmaPlot 11). The data were submitted to the Shapiro-Wilk normality test. The results were described as frequency, median, and range. ANOVA on ranks Tukey test and Mann Whitney were used for comparison within each group and between the groups, respectively. The two groups (pCMBs and control) were compared based on signalment characteristics (breed size, age, and sex) and intracranial and aged-related MRI findings. The four most prevalent breeds were compared to the control group and to the same breed size group (small, medium, or large breed groups). Additionally, age comparisons between sexes and breed sizes were performed to evaluate the longevity hypothesis. Contingency tables were generated for the categorical variables. The distribution of factors was compared between groups using the Chi-square (X²) test. The odds ratio (OR) and 95% confidence interval (CI) were determined for each comparison. Pearson’s correlation test and Spearman’s correlation test were used to assess the relationship between the number of pCMBs, and age as well as the presence of pCMBs and brain atrophy, respectively. P-values ≤0.05 were considered statistically significant.

## Results

A total of 1118 dogs underwent brain MRI. Among them, six were excluded due to incomplete radiology records, 309 due to lack of the T2* GRE sequence, and 56 cases corresponded to follow-ups. Seven hundred and forty-seven dogs met the inclusion criteria and were included in this study. The median age of the total population was ten years (range, 0–18 years). There were 58.9% (440/747) dogs ≧ 9 years, 35.6% (266/747) between 2 and 9 years, and 5.5% (41/747) <2 years. There were 386 (51.7%) males (209 neutered and 132 intact) and 361 (48.3%) females (258 spayed and 71 intact). Neuter status was not available for 77 animals (these were recorded as status unknown, not as unneutered). A total of 56 breeds were represented; the most common breeds were Mixed-breed dogs (146; 19.6%), French Bulldogs (82; 11%), Yorkshire Terriers (69; 9.2%), and Poodles (48; 6.4%). Among the pure breeds in the sample population, most of the dogs were small-sized breeds (55.4%) (Table 1).

**Table 1:** Signalment characteristics and results of univariate analysis of 747 dogs that underwent brain MRI from January of 2019 to September of 2020, Rio de Janeiro, Brazil.

Characteristics	All n(%)	pCMB n(%)	Control n(%)	OR	95% CI	p-value
Animals	747 (100%)	142 (19.0%)	605 (81.0%)	–	–	–
**Size**						0.02
Small (<15 kg)	414 (55.4%)	91 (64.1%)*	323 (53.4%)	1.56	1.07-2.27	0.03
Medium (15–30 kg)	100 (13.4%)	15 (10.6%)	85 (14.0%)	0.72	0.40-1.29	0.33
Large (>30 kg)	87 (11.6%)	9 (6.3%)*	78 (12.9%)	0.46	0.22-0.94	0.04
Mixed breed	146 (19.6%)	27 (19%)	119 (19.7%)	0.91	0.57-1.46	0.80
**Breeds**						0.04
Poodle	48 (6.4%)	22 (15.5%)	26 (4.3%)	4.08	2.24-7.45	<0.001
Yorkshire	69 (9.2%)	18 (12.7%)	51 (8.4%)	1.58	0.89-2.79	0.16
Shih Tzu	46 (6.1%)	10 (7.0%)	36 (6.0%)	1.45	0.69-3.04	0.43
Maltese	40 (5.3%)	10 (7.0%)	30 (5.0%)	1.20	0.58-2.47	0.77
**Age (years)**	9.3 ± 4.5; 10[<1-18]	13.6 ± 2.4; 14 [3-18]	8.3 ± 4.1; 9[<1-18]	--	--	<0.001
Young (<2ys)	41 (5.5%)	0 (0%)*	41 (6.8%)	0	0	<0.01
Adult (2–8ys)	266 (35.6%)	5 (3.5%)*	261 (43.1%)	0.05	0.02-0.12	<0.001
Elderly (≧9ys)	440 (58.9%)	137 (96.5%)*	303 (50.1%)	27.31	11.03-67.61	<0.001
**Sex**						0.03
Male	386 (51.7%)	62 (43.7%)*	324 (53.6%)	0.67	0.47-0.97	0.04
Female	361 (48.3%)	80 (56.3%)*	281 (46.4%)	1.49	1.03-2.15	0.04

pCMB: presumptive cerebral microbleeds; OR: odds ratio; CI: confidence interval; X² test *Statistically significant difference between pCMB and control groups.

**Fig 1:**
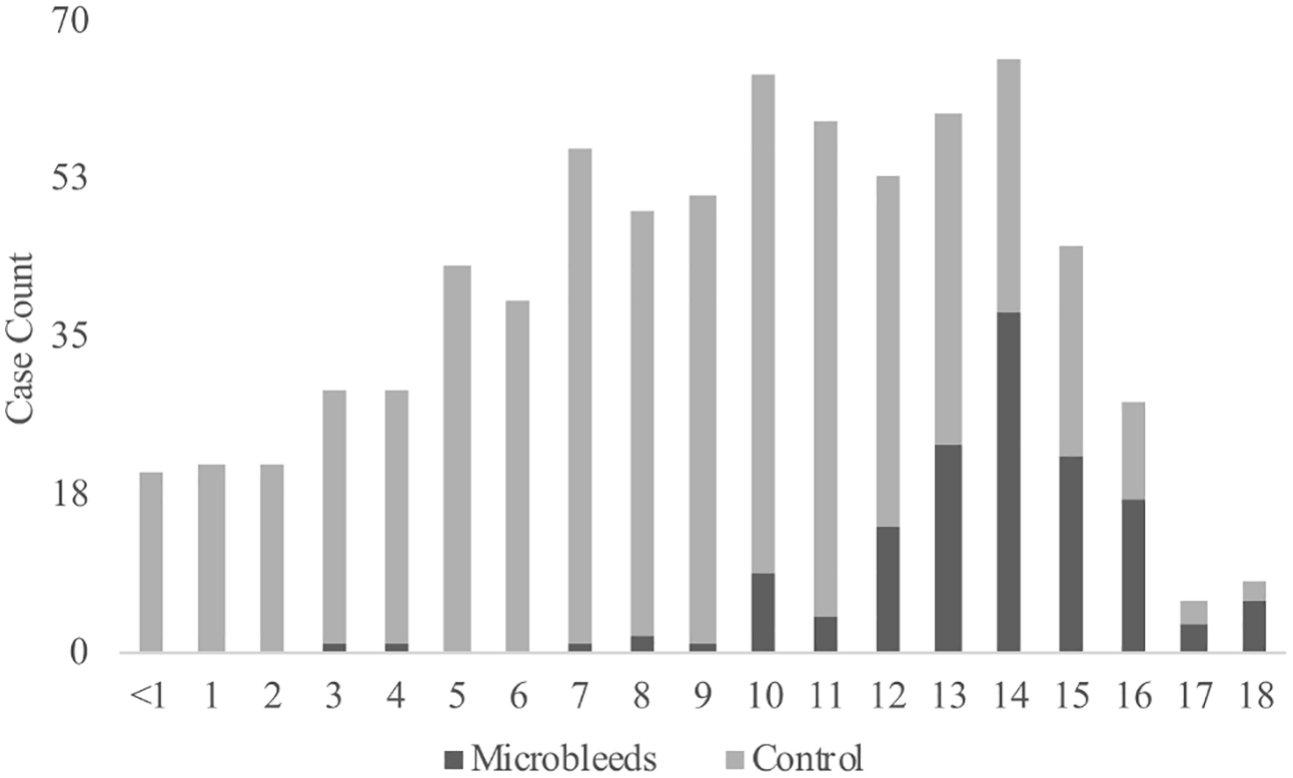
Distribution of presumptive cerebral microbleeds (pCMBs) identified in the population of dogs by age. Light gray bars represent dogs without pCMB (control) and dark gray bars represent dogs with pCMBs.

A total of 142/747 (19%) dogs had ≧ 1 presumptive cerebral microbleeds (pCMBs) and were included in the pCMBs group; the remaining 605/747 dogs were used as control cases. The detailed signalment characteristics distribution of each group is described in Table 1. Dogs with pCMBs were older than control dogs (14 [3–18] vs. 9 [0–18] years; p < 0.001). Almost all dogs (137/142, 96.5%) in the pCMBs group were older than 9. Statistical analyses showed that aged dogs were associated with higher odds of pCMBs, with dogs ≧9 years being significantly more affected than dogs <9 years (p < 0.001; OR 27.31; CI 11.03–67.60). Also, when plotted against age, it was found that the prevalence of pCMBs increased with age (p = 0.02), especially in those older than ten years (p < 0.001) (Fig 1).

The presence of pCMBs was different between sexes, with a higher prevalence in female than male dogs (80/142, 56.3% vs. 62/142, 43.7%; p < 0.033) (Table 1). Age analysis revealed that females were significantly older than males in the total population (median 11 vs 9 years, p < 0.05).

Concerning size, when mixed-breed dogs were included in the analysis, there was a tendency (p = 0.05) of difference. However, when those were excluded, it was found that small-breed dogs were significantly more affected compared to large-breed dogs (91/414, 64.5% vs. 9/87, 6.4%, p = 0.01). Age analysis showed that small breeds were significantly older than large breeds (median 10 vs 8 years, p < 0.01), The most frequent breeds represented in the pCMBs group included mixed-breed (27/146, 18.5%), Poodles (22/48, 45.8%), Yorkshire Terriers (18/69, 26.1%), Shih Tzu (10/46, 21.7%) and Maltese (10/40, 25%). When they were compared to the same breed population in the control group, only poodles were statistically different in prevalence of pCMBs compared to the other breeds (45.8% vs. overall 19%, p = 0.04) (Table 2).

**Table 2:** Distribution and results of univariate analysis to evaluate the association of presumptive cerebral microbleeds (pCMBs) with size and breed in 747 dogs that underwent brain MRI, from January of 2019 to September of 2020, Rio de Janeiro, Brazil.

Characteristics	All (n)	pCMB n(%)	Control n(%)	p-value*
**Size**	747 (100%)	142 (19.0%)	605 (81.0%)	0.022
Small	414 (55.4%)	91 (22%)ᵃ	323 (78%)	–
Medium	100 (13.4%)	15 (15%)ᵃᵇ	85 (85%)	0.121
Large	87 (11.6%)	9 (10.3%)ᵇ	78 (89.7%)	0.013
**Breed**				0.041
Poodle	48 (6.4%)	22 (45.8%)ᵃ	26 (54.2%)	–
Maltese	40 (5.3%)	10 (25%)ᵇ	30 (75%)	0.043
Shih Tzu	46 (6.2%)	10 (21.7%)ᵇ	36 (78.3%)	0.014
Yorkshire	69 (9.2%)	18 (26.1%)ᵇ	51 (73.9%)	0.027

^ab^Different letters indicate statistically significant differences *X² test.

The majority of dogs (88/142, 62%) had multiple lesions (>1 pCMBs). The number of pCMBs was significantly associated with age, with a higher number of lesions in older dogs (p < 0.001; r = 0.22). pCMBs were identified in all brain regions except brainstem (midbrain, pons, and medulla oblongata) and were mostly located in the cerebral cortex (90/142, 63.8%). Specifically, no lesions were identified in the medulla oblongata, while occasional lesions were seen in midbrain and pons regions. Most of the dogs (82/142, 57.7%) had a lobar distribution of the lesions (limited to the cerebral cortex or cerebellum) and 37/142 (26.1%) had pCMBs located in both lobar and deep regions (mixed distribution), while 23/142 (16.2%) had pCMBs identified in deep locations alone (Table 3).

**Table 3:** Distribution of presumptive cerebral microbleeds by anatomical location and number in 142 dogs with pCMBs.

Characteristic	n (%)
**Number of lesions**	
Single lesion	median 11 vs 9 years, p < 0.05 (38.0%)
Multiple lesions (2–5)	58 (40.8%)
Multiple lesions (>5)	30 (21.1%)
**Anatomical location**	
Cerebral cortex	90 (63.4%)
Basal nuclei	25 (17.6%)
Thalamus	18 (12.7%)
Cerebellum	15 (10.6%)
Midbrain	8 (5.6%)
Pons	3 (2.1%)
Medulla	0 (0%)
**Distribution pattern**	
Lobar only	82 (57.7%)
Deep only	23 (16.2%)
Mixed (lobar + deep)	37 (26.1%)

In the pCMB group, concurrent intracranial abnormalities based on MRI diagnosis were identified in 131/142 (92.3%) of dogs. Brain atrophy was found with higher frequency (87/142, 61.3%), followed by intracranial neoplasia (35/142, 24.6%), cerebrovascular diseases (24/142, 16.9%) and inflammatory diseases (3/142, 2.1%). Eleven dogs (11/142, 7.7%) had no other abnormalities on MRI. Compared to the control group, statistical analyses showed that the presence of pCMBs was significantly associated with brain atrophy (p < 0.001; r = 0.36) and cerebrovascular disease (p = 0.002; r = 0.14). Although statistically different, the correlation was weak. There was no association between pCMBs with the other MRI abnormalities (Table 4).

**Table 4:** Distribution and results of univariate analysis to evaluate the association of presumptive cerebral microbleeds (pCMBs) and other intracranial MRI findings in 747 dogs submitted to brain MRI, from January of 2019 to September of 2020, Rio de Janeiro, Brazil.

MRI Findings	pCMBs (n = 142)	Control (n = 605)	p-value*	R
Brain atrophy	87 (61.3%)	82 (13.6%)	<0.001	0.36
Cerebrovascular disease	24 (16.9%)	41 (6.8%)	<0.01	0.14
Intracranial neoplasia	35 (24.6%)	173 (28.6%)	0.34	–
Inflammatory disease	3 (2.1%)	76 (12.6%)	0.001	−0.13
Normal brain MRI	11 (7.7%)	107 (17.7%)	<0.01	−0.11

*X² test; R: correlation coefficient.

A total of 440 dogs ≧9 years were included in the analysis of the age-related MRI changes (Table 5). There were 136 dogs with MRI diagnosis of brain atrophy with no other concurrent abnormality. Of these, 48.9% (67/137) of the dogs were in the pCMB group while 22.8% (69/303) of the dogs were in the control group (p < 0.001).

**Table 5:** Age-related MRI changes in 440 elderly dogs (≧9 years) with and without presumptive cerebral microbleeds.

Characteristic	pCMB group (n = 137)	Control group (n = 303)	p-value
Brain atrophy only	67 (48.9%)	69 (22.8%)	<0.001
Interthalamic adhesion height (mm)	3.88 ± 0.13	3.99 ± 0.14	0.53
Leukoaraiosis	42/67 (62.7%)	37/69 (53.6%)	0.91

Mean value of interthalamic adhesion height (thickness) were 3.88 ± 0.13 mm and 3.99 ± 0.14 mm in the pCMB group and control group, respectively and they were not significantly different from each other (p = 0.53). The presence of periventricular white matter hyperintensities (leukoaraiosis) in T2W/FLAIR images were identified in 79/136 (58%) of these dogs (42 dogs in the pCMB group and 37 dogs in the control group), but there was no difference between the groups (p = 0.91).

**Fig 2:**
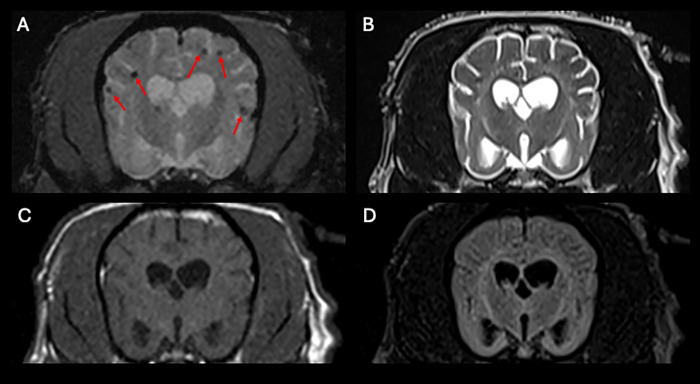
Representative MRI images showing typical presumptive cerebral microbleeds (pCMBs). **(A)** T2*-weighted gradient-recalled echo (GRE) transverse image showing multiple small round hypointense lesions (arrows) in the cerebral cortex with characteristic blooming artifact. **(B)** T2-weighted image of the same slice showing the lesions are not hyperintense. **(C)** T1-weighted image demonstrating absence of hyperintensity in the same regions. **(D)** FLAIR image confirming the lesions do not show hyperintensity characteristic of other pathologies.

Of the five dogs aged 3–9 years with pCMBs, three were female small breed dogs (two Poodles, one Yorkshire Terrier), one male medium breed (mixed), and one female large breed (mixed). All had multiple lesions and concurrent brain abnormalities, but numbers were too small for statistical analysis. Fig 2 illustrates typical pCMBs on MRI.

## Discussion

The results of this study confirm that presumptive cerebral microbleeds are an age-related neuroimaging finding increasingly recognized in dogs undergoing brain MRI, with an overall prevalence of 19%. It also demonstrated that small-breed dogs and females are significantly more affected, with an increasing tendency for pCMBs to occur with aging, especially in those older than 10 years.

Cerebral microbleeds are widely recognized in humans; however, there are only a few case series that report pCMBs in dogs [5,21–23], and only one recent study has evaluated their epidemiological aspects [3]. The most comprehensive study to date [3] evaluated 548 dogs and found a 9.3% prevalence of pCMBs, with an association to shorter life expectancy. However, that specific study used a 3T MRI and its sample was composed of a younger population (median age 7 years), compared to the current investigation. Despite similar designs and sample populations, a much higher prevalence of pCMBs (19%) was found in the current study compared to the previous study (9.3%) [3]. This difference may be attributed to several factors: (1) the older population of the current study compared to the previous study (median, 10 [0–18] years vs. 7 [0–19] years), (2) different MRI field strength (1.5T vs 3T), (3) different geographic populations (Brazilian vs North American), and (4) larger sample size (747 vs 548 dogs) allowing detection of more subtle prevalence differences. In people, prevalence rates also differ widely across reports, depending on the heterogeneity in the age of the population studied [10,13,24–26]. Additionally, the increase in the prevalence of pCMBs with age was confirmed, the same as occurs in humans [27,28], and the number of pCMBs was also associated with age. Multiple microbleeds related to aging are also commonly reported in elderly people [10].

The presence of pCMBs was most frequent in female and small-breed dogs. Despite the majority of dogs in the population being males (51.3%), the risk of pCMBs was higher in females (p = 0.04; OR 1.5; CI 1.03–2.15). In recent case series studies, 4/6 dogs reported with pCMBs were females [5,22,29]. However, a sex preference has not been reported yet. Indeed, a study involving 548 dogs [3] found no significant differences in the pCMBs related to sex. Our analysis showed that females were significantly older than males in our population (median 11 vs 9 years, p < 0.05), supporting the hypothesis that this finding may be explained by the longer median lifespan of females [30]. Similarly, smaller breeds’ median lifespan is longer than larger breeds [31,32], which may explain why small dogs were significantly more affected with pCMBs, since they live long enough to develop lesions associated with aging. Our data confirmed this hypothesis, as small breeds were significantly older than large breeds (median 10 vs 8 years, p < 0.01).

Among individual breeds, Poodles showed remarkably high prevalence of pCMBs (45.8% vs 19% overall, p < 0.001), which cannot be explained by age alone and suggests potential breed-specific susceptibility. This finding warrants further investigation, as Poodles are known for their longevity but may also have genetic predispositions to cerebrovascular changes. Yorkshire Terriers (26.1%), Shih Tzu (21.7%), and Maltese (25%) also showed higher than average prevalences, though not statistically significant, possibly due to smaller sample sizes.

Most of the presumptive cerebral microbleeds identified in this study were multiple and mainly located in the cerebral cortex, primarily at the gray-white matter junction, as has been previously reported [5,21,33]. In humans, this lobar distribution of CMBs is characteristic of CAA, a small vessel disease commonly seen in elderly people caused by the accumulation of B-amyloid within the cerebral blood vessels. In contrast, deep CMBs are associated with hypertensive arteriopathy [17,34–36]. Pathologic studies have shown similar features of CAA in aged dogs [37–40] and have found the presence of cerebral microhemorrhages in these cases [41–43]. One recent report on aged dogs found an association between CAA and perivascular microhemorrhages, with dogs with CAA being nine times more likely to have these associated microhemorrhages [43]. Although there are no studies in dogs comparing the MRI distribution of microbleeds with histopathologic correlation with CAA, given the similarities in amyloid distribution between people and dogs, a similar association seems likely. One study suggested that pCMBs might be a manifestation of CAA and represent a distinct degenerative brain disorder with some similarities to cognitive dysfunction [23]. To date, there are no studies in dogs investigating the clinical relevance of CMBs; however, shorter survival time was associated with these lesions [33] and, therefore, may not be incidental findings, similar to what is observed in humans.

Compared to the other concurrent intracranial findings identified on MRI, the presence of pCMBs in the sample population of the present study was significantly associated with brain atrophy. In people, cerebral microbleeds are commonly seen with brain atrophy and are present in the general elderly population [44–46]. A recent study has shown that old dogs with CMBs also had brain atrophy [23]. Because the current study lacks clinical data regarding the neurological status of the dogs, the clinical relevance of these findings remains unclear. Therefore, it is possible to hypothesize that the current study’s association may reflect aging characteristics since both brain atrophy and cerebral microbleeds are more likely to occur with age.

Indeed, even when only elderly dogs (≧9 years) were evaluated in this study, almost half of the dogs in the pCMB group had only brain atrophy, with no other concurrent abnormality (vs 22.8% in the control group). The median interthalamic adhesion height measurements were smaller but not statistically different in elderly dogs (pCMB group = 3.88 mm; Control group = 3.99 mm, p = 0.53). This measurement has been suggested as a valuable method for evaluating brain atrophy in dogs. An interthalamic adhesion thickness of <5mm has been proposed to define brain atrophy [1,47]. Another MRI feature of aging found in the present study was the presence of bilateral periventricular white matter lesions in 79/136 (58%) of the dogs with brain atrophy. These lesions are also commonly seen in elderly people and have been associated with brain atrophy and cerebral microbleeds in MRI studies of aging in humans [48–50]. A recent study in aged dogs found that 10/14 dogs with brain atrophy also had these periventricular white matter lesions and introduced leukoaraiosis as a descriptive term for this MRI feature [4]. Although this may be part of the normal aging process, there is increasing evidence that leukoaraiosis may have clinical implications in humans [51]. However, to date, there are no clinicopathological studies in dogs investigating the clinical significance of these lesions.

The main limitations of this study include several important factors that must be considered when interpreting the results. First and most importantly, this study is based entirely on “presumptive MRI diagnoses” without histopathological confirmation. All diagnoses of pCMBs and concurrent conditions should be considered presumptive, and no assumptions about clinical relevance can be made from this study. Second, clinical and neurological status of the dogs was not recorded due to the retrospective nature of the study, preventing assessment of the clinical significance of the findings. Third, mixed-breed dogs were not subdivided by size categories, which may have influenced the statistical analysis results and could have enhanced the power of breed-size comparisons. Fourth, the absence of susceptibility-weighted imaging (SWI) represents a technical limitation. SWI has been used for CMBs detection due to its higher sensitivity compared to T2* GRE, especially for quantifying the lesions [52]. Besides, SWI also allows better differentiation of microbleeds from calcifications [53,54], which can result in a similar appearance on standard T2* GRE sequences. Fifth, the study lacks data on systemic comorbidities such as hypertension, cardiac disease, and renal disease, which could provide important insights into the pathophysiology of pCMBs. Finally, the retrospective design limited data availability and standardization of imaging protocols. Although SWI was unavailable in this study, all imaging characteristics were consistent with previous reports. Therefore, the lesions observed on MRI were presumptive cerebral microbleeds.

Despite these limitations, this study provides valuable epidemiological data on pCMBs in a large canine population and suggests that these lesions are more common than previously reported. The strong association with aging, female sex, and small breed size provides important baseline data for veterinary neurologists and may guide future prospective studies investigating the clinical significance of these findings.

Presumptive cerebral microbleeds are a common age-related MRI finding in small-breed dogs and females, with a 19% prevalence in dogs undergoing brain MRI. They are frequently associated with brain atrophy and show increasing prevalence in dogs older than ten years. Breed-specific differences, particularly the high prevalence in Poodles, warrant further investigation. Given the similarities between people and dogs, further studies are needed to investigate the histopathologic correlation with the imaging findings to better understand their clinical relevance and potential impact on cognitive function and survival.
